# Identification of the m^6^A RNA Methylation Regulators WTAP as a Novel Prognostic Biomarker and Genomic Alterations in Cutaneous Melanoma

**DOI:** 10.3389/fmolb.2021.665222

**Published:** 2021-07-05

**Authors:** Zi-Yi Feng, Ting Wang, Xin Su, Shu Guo

**Affiliations:** Department of Plastic Surgery, The First Affiliated Hospital of China Medical University, Shenyang, China

**Keywords:** cutaneous melanoma, m6A regulators, WTAP, biomarker, survival analysis, genomic alterations

## Abstract

**Background:** The purpose of our research was to establish a gene signature and determine the prognostic value of m^6^A methylation regulators in cutaneous melanoma and WTAP as a protective gene in cutaneous melanoma prognosis, we also evaluated gene mutations in cutaneous melanoma.

**Methods:** We downloaded the RNA-seq transcriptome data and the clinical information for cutaneous melanoma patients from the GTEx and TCGA databases. Consensus clustering analysis was applied to divide the samples into two groups. Then the least absolute shrinkage and selection operator (LASSO) analyses were conducted to construct a risk signature, and we use external and internal datasets to verify its predictive value. We further searched the cBioPortal tools to detect genomic alterations and WTAP mutations. Finally, WTAP was further identified as a prognostic factor, and the related mechanisms mediated by WTAP were predicted by gene set enrichment analysis (GSEA). Experimental validations and have been further carried out.

**Results:** Notably, m^6^A RNA methylation regulators play significant roles in tumorigenesis and development. In total, we selected three subtypes of cutaneous melanoma according to consensus clustering of the m^6^A RNA methylation regulators, and the stage of cutaneous melanoma was proven to be related to the subtypes. The Cox regression and LASSO analyses built a risk signature including ELF3, ZC3H13 and WTAP. The prognostic value of the risk signature in internal and external datasets have been proven then. The whole-genome and selected gene WTAP mutations were further explored. WTAP as a single prognostic factor was also explored and found to serve as an independent protective prognostic factor.

**Conclusions:** Our study constructed a stable risk signature composed of m^6^A RNA methylation regulators in cutaneous melanoma. Moreover, WTAP was identified as a valuable prognostic factor and potential molecular target for cutaneous melanoma treatment.

## Introduction

As one of the deadliest forms of cancer, cutaneous melanoma is responsible for more than 75% of deaths among all cutaneous cancers and no less than 5% of cases of cutaneous cancer (Rebecca et al., 2020). Approximately 100,350 new cases were estimated in the US in 2020, associated with 6,850 deaths, and there is a 2.5–3.6% lifetime probability of developing the disease (Siegel et al., 2020). Cutaneous melanoma refers to the malignant transformation of melanocytes, a type of cell that produces melanin and also regulates the absorption of ultraviolet radiation (UVR) and skin pigmentation (Lin and Fisher, 2007). The malignant transformation of melanocytes has a high mutational burden and is linked with NRAS, HRAS, BRAF, neurofibromin 1 (NF1), KIT, GNAQ, and cyclin-dependent kinase inhibitor 2A (CDKN2A) mutations (Curtin et al., 2005; Bastian, 2014). Known for its high levels of aggressiveness and therapy resistance, cutaneous melanoma was considered to be untreatable before several checkpoint inhibitor therapies were approved, which later proved to show remarkable improvements. Nevertheless, approximately 70% of cutaneous melanoma patients and their attending physicians still face the challenges of immune checkpoint inhibitor (ICI) resistance, high mortality rates, recurrence, and dissemination (Jerby-Arnon et al., 2018). Consequently, attention to the underlying molecular mechanisms underlying this malignancy and the exploration of additional novel targets of cutaneous melanoma treatment is greatly needed.

In recent years, research on epigenetics in RNA has begun to increase in various fields. Posttranscriptional regulatory events such as RNA methylation have attracted increasing attention from researchers and have recently been identified as a mechanism for regulating tumorigenesis. The modification of RNA bases, especially RNA methylation, is being gradually understood for its effects in encouraging RNA translation, metabolism, splicing and stability (Lan et al., 2019). Among them, m^6^A RNA methylation has been a new research hotspot as the most frequent RNA modification in eukaryotes, Having been recognized as the most abundant and conserved internal transcriptional modification, N6-methyladenosine (m^6^A) has the ability to alter cancer cell initiation, tumor invasion, proliferation, differentiation inhibition, metastasis and therapy resistance (Wang et al., 2020). m^6^A refers to the methylation of the nitrogen atom (N) at the 6th position of adenine; enzymes can “write,” “erase” or “read” the methyl group and thus regulate the RNA. Methyltransferase complexes can be divided into three categories: “writers” refers to methyltransferases, mainly including METTL3, METTL14, WTAP, etc., which transfer the methyl to the nitrogen atom (N) at the sixth position of adenine; “erasers” refers to demethylases, mainly including FTO, ALKBH5, etc.; and “readers” refers to specific RNA binding proteins that can bind the DNA m^6^A modified RNA and have specific biological functions, including YTHDC1, YTHDC2, YTHDF1, YTHDF2, and so on. Therefore, the methylation of RNA in cells is reversible and dynamic. After methylation, RNA is transported out of the nucleus into the cytoplasm and is recognized and bound by downstream methylated reading proteins to regulate the function of the RNA (Nombela et al., 2021).

**FIGURE 1:**
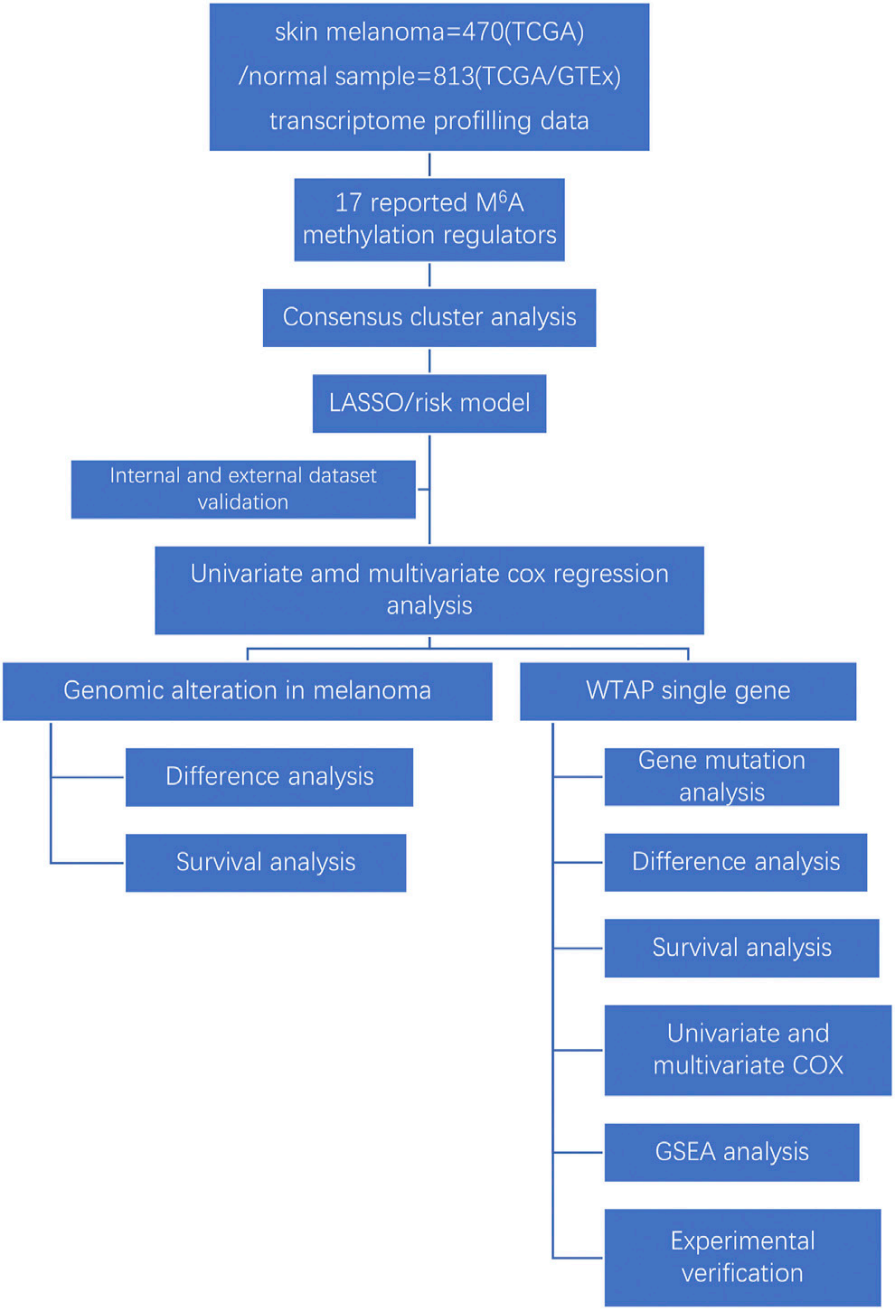
Workflow chart of data generation and analysis.

Recent studies have shown remarkable progress in linking m^6^A RNA demethylation with fat mass and obesity-associated protein (FTO) (Yang et al., 2019)and ALKBH5 (Li et al., 2020), which is strongly associated with cutaneous melanoma development and reactions to cancer therapy. Notably, other studies also revealed that m^6^A RNA methylation is significantly related to uveal cutaneous melanoma (UM) and conjunctival cutaneous melanoma (CM) progression and migration by promoting HINT-2 translation Jia et al. (2019) and the regulatory enzyme METTL3 targeting c-Met (Luo et al., 2020), thus demonstrating the close relationship between m^6^A RNA alterations and cutaneous melanoma. Wilms’ tumor 1-associating protein (WTAP) is widely expressed in the nucleus and functions as a putative splicing regulator. It was first recognized for its interaction with Wilms’ tumor 1 (WT1), which is quintessential in the urogenital system. Notably, WTAP is necessary for the cell cycle progress by stabilizing cyclin A2 mRNA and mammalian early embryo development Horiuchi et al. (2013), and it also has important functions in cell behaviors and cancer development (Yu et al., 2019). It was recently suggested that WTAP may be correlated with numerous types of cancers (Zhang et al., 2016), but there is little knowledge about the role of WTAP in cutaneous melanoma or its underlying mechanism. Our study is the first to investigate the difference in WTAP expression between cutaneous melanoma and normal tissue and its connection to clinicopathological characteristics, as well as to predict its mechanism of action. Our findings may provide strategies for exploring new therapeutic targets of cutaneous melanoma and may have significance for improving the clinical outcomes and prognosis of cutaneous melanoma (Figure 1).

## Materials and Methods

### Data Processing

The RNA transcription information of 471 cutaneous melanoma samples and 1 normal sample and the corresponding clinicopathological information (Table 1) of 470 cutaneous melanoma patients were downloaded from The Cancer Genome Atlas (TCGA) database (http://cancergenome.nih.gov/). To obtain more samples for the control group, that is, normal tissue, we downloaded the RNA-seq transcriptome data of 812 normal human cutaneous tissues from the GTEx database (https://www.gtexportal.org/home/datasets). The external validation dataset is obtained from the Gene Expression Omnibus (GEO) database (Lin et al., 2021). The RNA transcriptome data were normalized by fragment per kilobase of exon model per million (FPKM, mean fragment per kilobase million (Leek, 2014)). First, we transformed the probe IDs into official gene symbols on the basis of the platform. The probe with the higher expression level was selected when multiple probe IDs matched to one gene symbol, then log2 (x + 1) is used to normalize the original matrix data conversion. The genomic alterations of WTAP were extracted from cBioPortal (www.cbioportal.org). The single nucleotide polymorphism (SNP) data for 471 cutaneous melanoma patients were also extracted from TCGA database by VarScan2 Variant Aggregation and Macutaneousg.

**TABLE 1:** The clinical characteristic information of the melanoma patients in TCGA (470 in total).

Characteristics	Number of cases	Percentages (%)
Age		
<=65	298	63.40
>65	164	34.9
Not available	8	1.70
Gender		
Male	290	61.70
Female	180	38.30
Survival status		
Alive	259	55.11
Dead	211	44.89
Stage		
I	77	16.38
II	140	29.79
III	171	36.38
IV	23	4.89
Not available	59	12.56
T Classification		
T0	23	4.89
T1	41	8.72
T2	78	16.60
T3	90	19.15
T4	143	30.43
Not available	95	20.21
N classification		
N0	235	50.00
N1	74	15.74
N2	49	10.43
N3	55	11.70
Not available	57	12.13
M Classification		
M0	418	88.94
M1	24	5.11
Not available	28	5.95

### Selection of m^6^A RNA Methylation Regulators and Correlation Detection

To make the research more comprehensive, we selected 17 classical genes (METTL3, METTL14, WTAP, RBM15, ZC3H13, YTHDC1, YTHDC2, YTHDF1, YTHDF2, HNRNPC, FTO, ALKBH5, KIAA1429, IGF2BP1, IGF2BP2, IGF2BP3, and ELF3) as m^6^A RNA methylation regulators extracted from previous studies (Luo et al., 2020; Tang et al., 2020). Gene expression were extracted from the TCGA-SCKM cohort and GTEx cutaneous group with corresponding clinical information. To better understand the differences in m^6^A RNA methylation regulators between cutaneous melanoma and normal samples, heatmaps and violin plots were made by using the “limma” R package (http://www.bioconductor.org/packages/release/bioc/html/limma.html). The screening conditions we used to define the differentially expressed genes were *p* < 0.05, *p* < 0.01 and *p* < 0.001 respectively. Additionally, “corrplot” R package was applied to draw the correlation between the 17 genes (https://cran.r-project.org/web/packages/corrplot/vignettes/corrplot-intro.html).

### Consensus Clustering Analysis

To observe the role of 17 m^6^A RNA methylation regulatory factors in the cutaneous melanoma cohort, consensus clustering analysis was then conducted, with a cumulative distribution function (CDF) for k = 2–9. The “ConsensusClusterPlus” package (1,000 iterations and resample rate of 80%, https://bioconductor.org/packages/ConsensusClusterPlus/) assigned the patients into two categories with 1,000 permutations random sampling algorithm. Principal component analysis was performed to better identified the three groups. The overall survival between clusters was calculated by Kaplan–Meier analysis. The heatmap painted by “pheatmap” R package (https://CRAN.R-project.org/package=pheatmap) showed the relationship between grouping and clinicopathological factors.

### Prognostic Signature Generation and Validation

Multivariate Cox regression was used to calculate the correlation of m^6^A RNA methylation regulator genes with overall survival in cutaneous melanoma. Risk ratios (HRs) were used as the risk factor evaluation criteria. When the HR is greater than 1, the gene is considered a risk factor. Otherwise, it is considered a protective factor. Meanwhile, the risk score was characterized by the coefficients on the basis of the result of the least absolute shrinkage and selection operator (LASSO) algorithm. Finally, based on the minimum criteria, three regulators along with their coefficients were chosen, the risk score was calculated by applying the following formula (Huang et al., 2020):

$$Risk\;score=\sum_{i=1}^{n}Codfi^{*}x_{i}$$

where Codfi is the coefficient and $x_{i}$ is the relative expression value of the selected regulator after transformation. Then, the cutaneous melanoma cohort was divided into a high-risk and low-risk group based on the cutoff value we calculated.

### Evaluating the Prognostic Value of the Signature in Internal and External Datasets

The overall survival between the high- and low-risk groups was estimated by Kaplan–Meier analysis with the log-rank test. Then the receiver operating characteristic (ROC) curve for patient survival was analyzed to assess the prognostic value. Similarly, the relationships among the clinical factors (age, sex, stage, T stage, N stage, M stage) was also visualized through a heatmap by the “pheatmap” R package. In order to further verify the validity of our signature, we validated using the GSE65904 with 214 melanoma samples dataset downloaded from the Gene Expression Omnibus (GEO) database. The signature calculated by the mentioned formula, and the Kaplan-Meier and ROC curve analysis were implemented in progress. Both univariate and multivariate Cox regression analyses were conducted to predict whether these factors can be served as independent prognostic factors in cutaneous melanoma patients.

### Analysis of Genomic Alterations and Related Gene Enrichment

The genes with the top 30 mutation rates in cutaneous melanoma samples were visualized by twoR packages, “GenomeInfoDbData” and “GenVisR” (https://cran.r-project.org/web/packages/viridis/index.html.). According to these 30 mutant genes, we divided those samples into mutation group and normal group to calculate overall survival**.** The mutations and putative copy number alterations of WTAP in melanoma were got from the cBioPortal tool (http://cbioportal.org) (Gao et al., 2013).

### Identifying the WTAP as an Independent Prognostic Factor

WTAP has been selected as an independent prognostic factor in cutaneous melanoma. First, “limma” R package was applied to visualize the WTAP expression differences in normal and tumor samples. According to the expression of WTAP, the data were divided into two groups and overall survival between two clusters were calculated. What is more, the Gene set enrichment analysis (GSEA) 4.1.0 was used to predict downstream access.

### Patient Samples and Immunohistochemistry

A total of seven human cutaneous melanoma tissues and their adjacent tissues were collected for immunohistochemical technique (IHC) from The First Hospital of China Medical University. Pathologists assessed the histological features of the specimens by referring to the standard criteria. The research was authorized by the Ethics Commission of the First Hospital of China Medical University (No. 2016-2-3) and was executed based on the ethical principles of the World Medical Association Declaration of Helsinki. Expression of WTAP gene in cutaneous melanoma and their adjacent tissues was assessed by immunohistochemistry (Proteintech, B600010) following the manufacturer’s instructions. In order to quantify WTAP expression, we first evaluated the staining intensity according to the following criteria: negative (score 0), weak (score 1), moderate (score 2), and strong (score 3). Meanwhile, the staining extent was graded into five levels: negative (score 0), 0–25% (score 1), 26–50% (score 2), 51–75% (score 3) and 76–100% (score 4). The merged overall score calculated by the intensity score multiply by percentage score.

### Cell Culture and Transfection

Human A375 cells were purchased from the Cell Bank of the Chinese Academy of Sciences (Shanghai, China) and cultured in DMEM medium (Gibico). 10% certified heat-inactivated fetal bovine serum (FBS; Gibico), penicillin (100 U/ mL), and streptomycin (100 mg/ ml) were supplied to culture cells. 37°C and a humidified 5% CO2 atmosphere were also apllied. Plasmids were constructed by Syngentech (Beijing China) based on the sequence of human WTAP in gene bank (NM_004906). The pZDonor-CMV-MCS-BGH_pA-hef1a-EGFP-P2A-neo vector was used for WTAP overexpression. Using Lipofectamine™ 3,000 Transfection Reagent (Thermo Fisher), cells were transfected with pc-WTAP (5 μg), or pc-Con (5 μg) as a negative control, based on the manufacturer’s instructions.

### Isolation RNA and Quantitative Real-Time PCR

Total RNA was extracted by using RNAiso Plus (Takara), and cDNA was generated by the PrimeScript RT Reagent Kit (Takara). Quantitative real-time PCR using Powerup SYBR Green PCR Master Mix (Life Technologies) was performed using a real-time PCR system (Applied Biosystems).

### Western Blotting

The cells were harvested and rinsed by PBS for three times after transfection for 48 h. Then the lysis buffer was used for the cell extracts and centrifuged at 13,000 xg for 30 min at 4°C. Sodium dodecyl sulfate–polyacrylamide gel electrophoresis (SDS-PAGE) was applied to separated protein samples then transferred to polyvinylidene fluoride membranes. 5% milk blocked samples for 1 h, then we used the primary antibody in 5% BSA overnight at 4°C to incubate. A secondary antibody labeled by horseradish peroxidase (Invitrogen) incubate the membrane after washing. Bio-Rad Imaging System for the visualization and quantification of the band signals.

### Cell Proliferation Assay

A375 cells were seeded in 96-well plates with the intensity of 10,000 cells per well. CellTiter 96 AQueous One Solution Cell Proliferation Assay (Promega) was used to assessed according to the manufacturer’s protocol after 24, 48, and 72 h.

### Flow Cytometry to Detect the Apoptosis and Cell Cycle

Apoptosis was assessed by the Annexin V:PE Apoptosis Detection Kit I (DOJINDO), following the manufacturer’s instructions. Then samples were detected by a flow cytometer (BD FACSCalibur).

For cell cycle analysis, cells were collected and pre-fixed in 75% cold ethanol and stored overnight at 4°C. After rinsing for 3 times in PBS and staining in propidium iodide (PI) for 30 min, cell cycle was detected by the flow cytometer (BD FACSCalibur).

### Migration Assays

Transwell assay evaluated the migration of the A375 melanoma cells. First we collected and resuspended 10,000 cells for the migration assay, cells were seeded into the upper chamber with an 8.0 μm pore (Corning, United States). While DMEM medium containing 20% FBS in the lower chambers. After incubating cells for 24 h, we removed the upper surface and the lower cells fixed with 100% methanol then the hematoxylin staining was performed and the observed under microscope, the above experiment was carried out three times and the number of migrated cells was counted repeatedly.

### Statistical Analysis

R software (Version 4.0.3) was utilized to calculate all statistical analyses, and the data presented as the means ± standard deviations. Differences between two groups analyzed by the Wilcoxon test, while the difference among multiple groups applied the one-way analysis of variance (ANOVA). The risk score was obtained by the coefficients which calculated by the LASSO algorithm. Moreover, Kaplan–Meier analysis and a log-rank test were used to calculated the differences in overall survival. Statistical significance was recognized at a P-value threshold of 0.05. In some cases, the false discovery rate was used to correct the p value and, items with corrected p-values of no more than 0.05 were taken.

## Results

### Differential Expression of m^6^A RNA Methylation Regulators in Normal Skin and Cutaneous Melanoma

**FIGURE 2:**
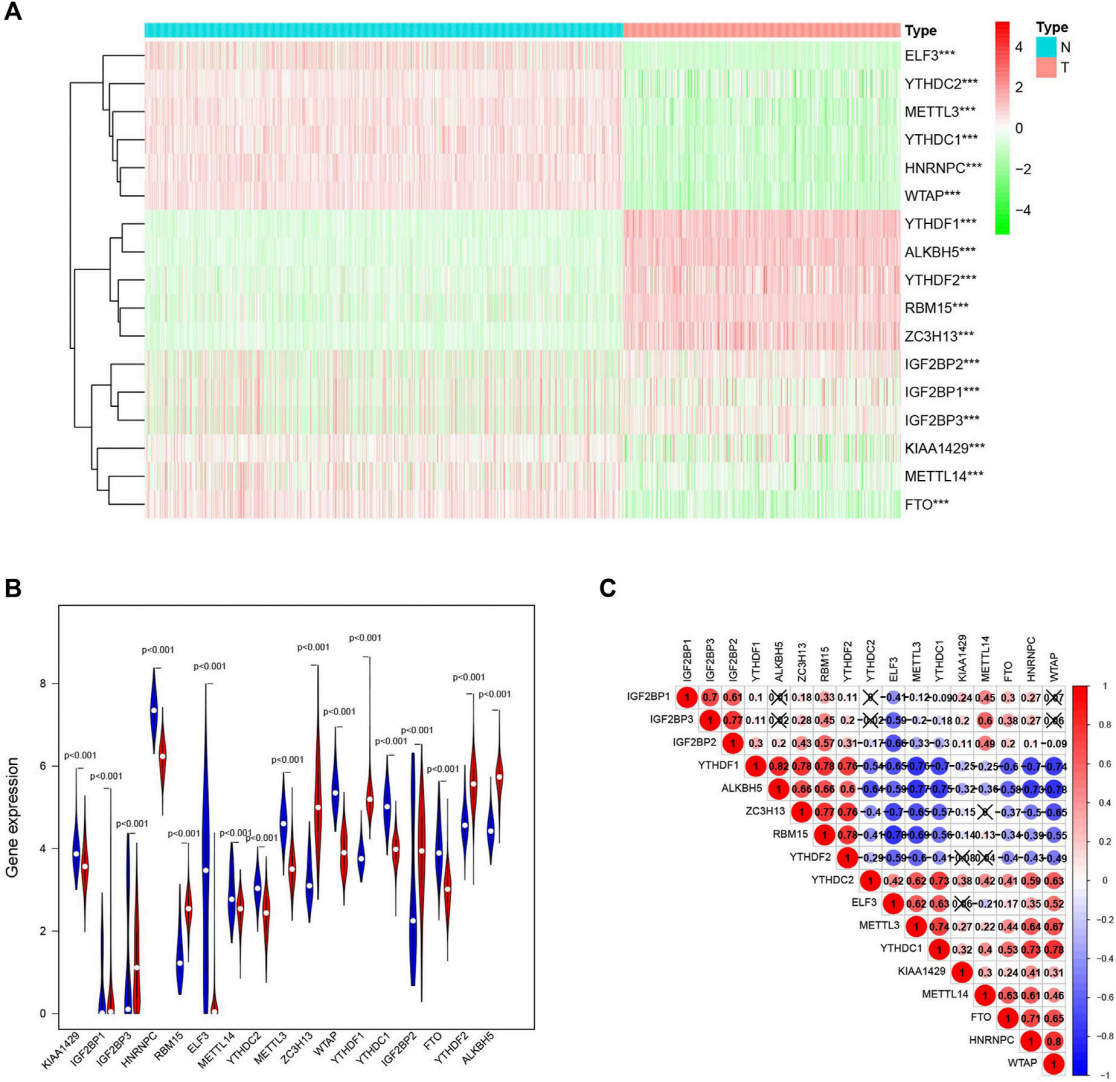
The expression characteristics and correlations of m^6^A RNA methylation regulators in melanoma. **(A)** Heatmaps presented the overall expression of 17 m^6^A RNA methylation regulators in melanoma tissues and normal tissues from The Cancer Genome Atlas (TCGA) and The Genotype-Tissue Expression (GTEx) datasets *p* < 0.05 (“*”), *p* < 0.01 (“**”), and *p* <0.001(“***”) **(B)** The differential expression of the m^6^A RNA methylation regulators was visualized by vioplot (blue means normal skin tissues, red means melanoma samples). **(C)** The interaction of the 17 m^6^A RNA methylation regulators.

It is noteworthy that the m^6^A RNA methylation regulators are necessary in the occurrence and development of cancer. To further understand the relationship, we systematically researched the transcription level of the 17 RNA regulators in TCGA datasets. To contribute to this discussion, a heatmap (Figure 2A) and a violin plot (Figure 2B) were used to show the difference. There was a significant difference in all 17 m^6^A RNA methylation regulators between normal skin and cutaneous melanoma, with all P values less than 0.001. As can be deduced from the picture, all 17 m^6^A RNA methylation regulators were split into two groups. One group (IGF2BP1, IGF2BP3, RBM15, ZC3H13, YTHDF1, IGF2BP2, YTHDF2, ALKBH5) were highly expressed in the cutaneous melanoma group, while the other group (KIAA1429, HNRNPC, ELF3, METTL14, YTHDC2, METTL3, WTAP, YTHDC1, and FTO) were highly expressed in the normal tissue.

To further explore the relationship among the 17 m^6^A RNA methylation regulators, a correlation between them was performed (Figure 2C). Among them, YTHDF1/ALKBH5 (r = 0.82) and WTAP/HNRNPC (r = 0.80) clearly had the most relevance.

### Consensus Clustering of m^6^A RNA Methylation Regulators Identified Two Clusters of Cutaneous Melanoma and Correlation With Clinicopathological Features

**FIGURE 3:**
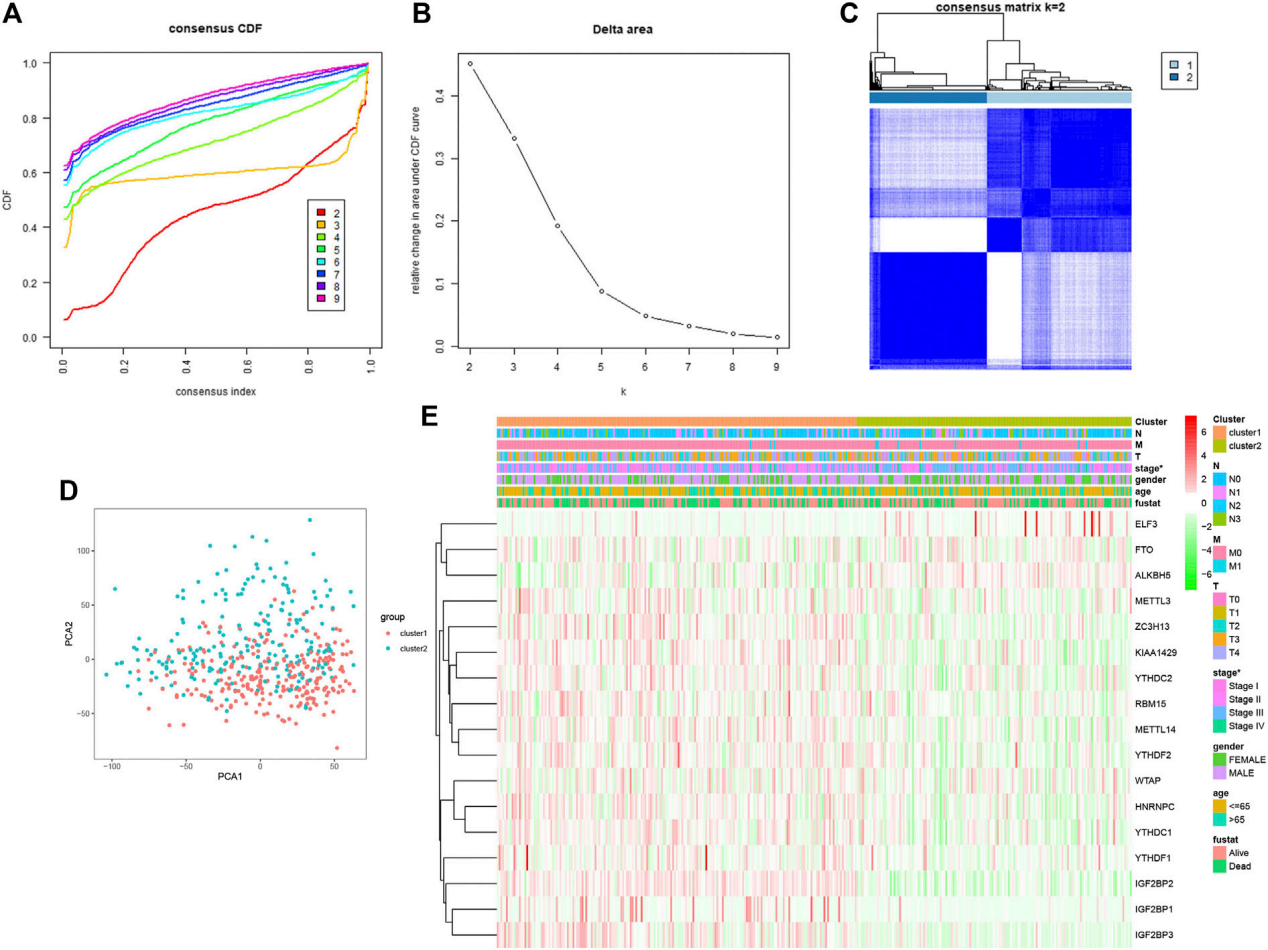
Association between the m^6^A RNA methylation regulators and clinicopathological and prognostic features of melanoma patients. **(A)** consensus clustering model with cumulation distribution function (CDF) for K = 2–9 (K means cluster count). **(B)** Relative change in area under the CDF curve for K = 2–9. **(C)** The Cancer G enome Atlas (TCGA) melanoma cohort was classified into three clusters with K = 2. **(D)** Principal component analysis of the total RNAexpression profile of cluster 1 (red), cluster 2 (blue). **(E)** The correlation of the two cluster with clinicopathologic features was visualized by heatmap. p < 0.05 (“*”).

To explore the connection between the m^6^A RNA methylation regulator expression profile and cutaneous melanoma prognosis, we grouped all 471 cutaneous melanoma cases in an unbiased way by using consensus clustering analysis. The stability of the clustering increased from k = 2 to 9, k means cluster count (Figures 3A,B), and K = 2 (Figure 3C) was selected as the most sensible choice. Moreover, we conducted principal component analysis (PCA) to further compare cluster 1 and cluster 2 (Figure 3D). However, there was no clear separation among the dots representing the two clusters or in the overall survival (Supplementary figure 1I) among the three clusters. We can thus conclude that there is no significant difference. As can be deduced from the heatmap (Figure 3E), only tumor stage was related to the clustering we established.

The possible reasons for the absence of significant differences may be that the clustering algorithm is not as sensitive as we expected or the size of the sample needed to be larger than we expected.

### Prognostic Value of the m^6^A RNA Methylation Regulators and the Construction of the Risk Model

**FIGURE 4:**
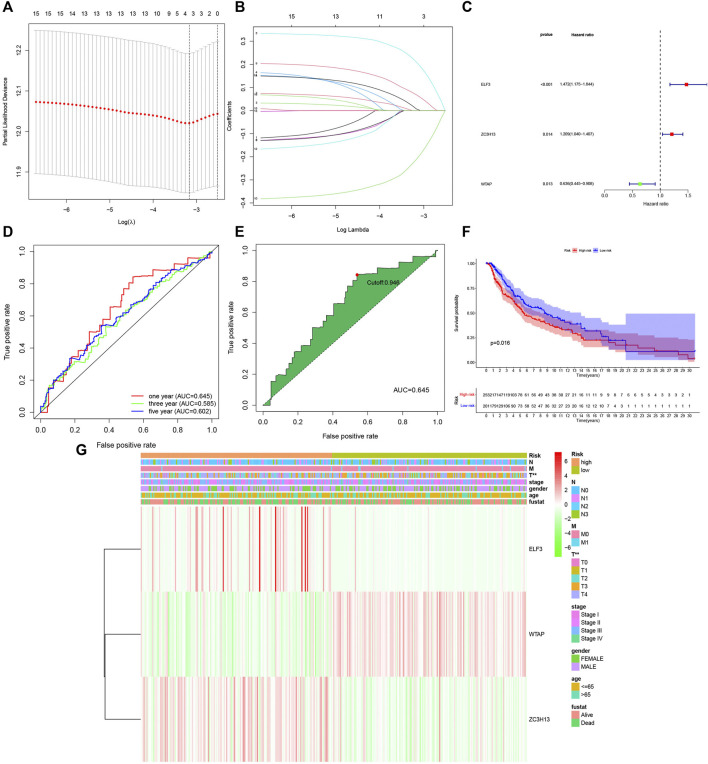
Construction of the three m^6^A RNA methylation regulators-based risk signature. **(A–B)** Determination of the number of factors by the LASSO analysis. **(C)** Multivariate analysis of the three-gene signature (ELF3,ZC3H13, AND WTAP). **(D)** Receiver operating characteristic (ROC) curves for 1-,3-,5-year prognosis. **(E)** 1 year was chosen to calculate the best cutoff value. **(F)** The kaplan-Meier curves of melanoma patients at the high-risk group and low-risk group in TCGA cohort. **(G)** The expression features of the three m^6^A RNA methylation regulators and the disteibution of clinicopathological features were compared between the low-and high group of The Cancer Genome Atlas (TCGA) melanoma datasets. *p* < 0.01 (“**”).

As different expression levels of the m^6^A RNA methylation regulators were observed in cutaneous melanoma and normal tissue, the prognostic value of the factors should be further explored. The LASSO algorithm, a generalized linear model, was constructed for further analysis of prognostic factors based on the expression of m^6^A RNA methylation regulators the in the TCGA cohort (Figures 4A,B). From this, three important m^6^A RNA methylation regulators were selected, which included ELF3, ZC3H13, WTAP, multivariate Cox regression (Figure 4C) indicated that “ELF3” and “ZC3H13” served as risk factors that were closely related to poor survival, while “WTAP” was a protective factor among cutaneous melanoma patients. These three we selected had significant correlation to survival (*p* < 0.1). Meanwhile, receiver operating characteristic (ROC) (Figure 4D) analysis was used to examine whether the model according to the risk score was sensitive and specific for prognosis. The area under the curve (AUC) values were accumulated to reflect the specific size according to the ROC curve. We can summarize from Figure 4D that the one year has the best effect. Therefore, we chose one year to calculate the most suitable cutoff value, and the sample was split into two groups, a high-risk group and a low-risk group with a 0.946 treated as the cutoff point (Figure 4E).

Meanwhile, we further estimated the overall survival (Figure 4F) between the two groups, and a significant difference was observed. A remarkable difference in overall survival was found between the high- and low-risk groups, with a 5-years survival rate of 86% in the low-risk group and 66% in the high-risk group, with a difference of 20%.

Hence, we compared the clinicopathological characteristics (including stage, sex, age, survival time, and TNM) of the groups with different risk scores. The heatmap (Figure 4G) clearly shows that there was a correction between the group assignment and clinicopathological factors (T and stage), with a P value less than 0.01.

**FIGURE 5:**
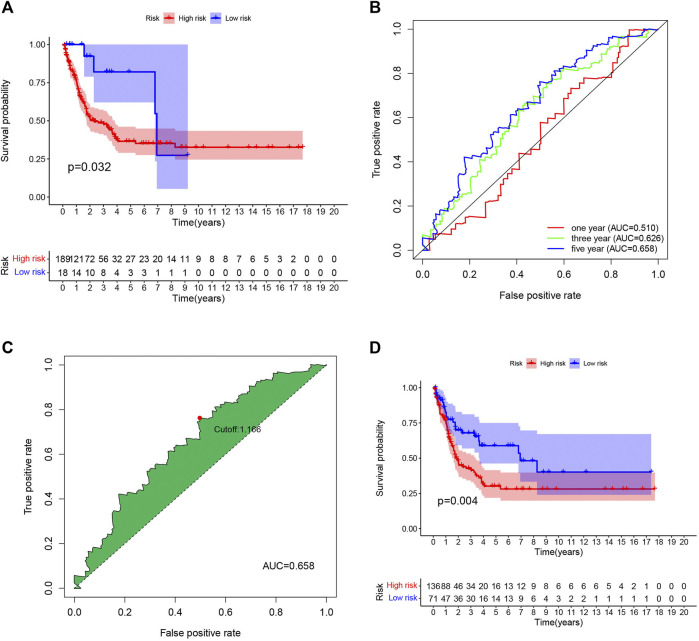
External validation of the risk signature. **(A)** The same cutoff value as TCGA was used to verify in GEO database. **(B)** Receiver operating characteristic (ROC) curves for 1-,3-,5-year prognosis in GEO database. **(C)** 5-year was chosen to calculated the brst cutoff value in GEO database. **(D)** The Kaplan–Meier curves of melanoma patients at the high group and low-risk group in GEO cohort.

To ensure the accuracy of the signature we constructed, we selected external data sets for verification. One GEO dataset (GSE65904) comprising 214 melanoma samples was used as a validation cohort. Consistent with our TCGA database results, the lower risk scores represented a higher possibility of survival in patients (Figure 5A). Then we calculate the cutoff value in GEO separately (Figures 5B,C), Similarly, the samples with low-risk scores showed longer overall survival times (*p* < 0.05), proved our model was feasible and accurate (Figure 5D).

**FIGURE 6:**
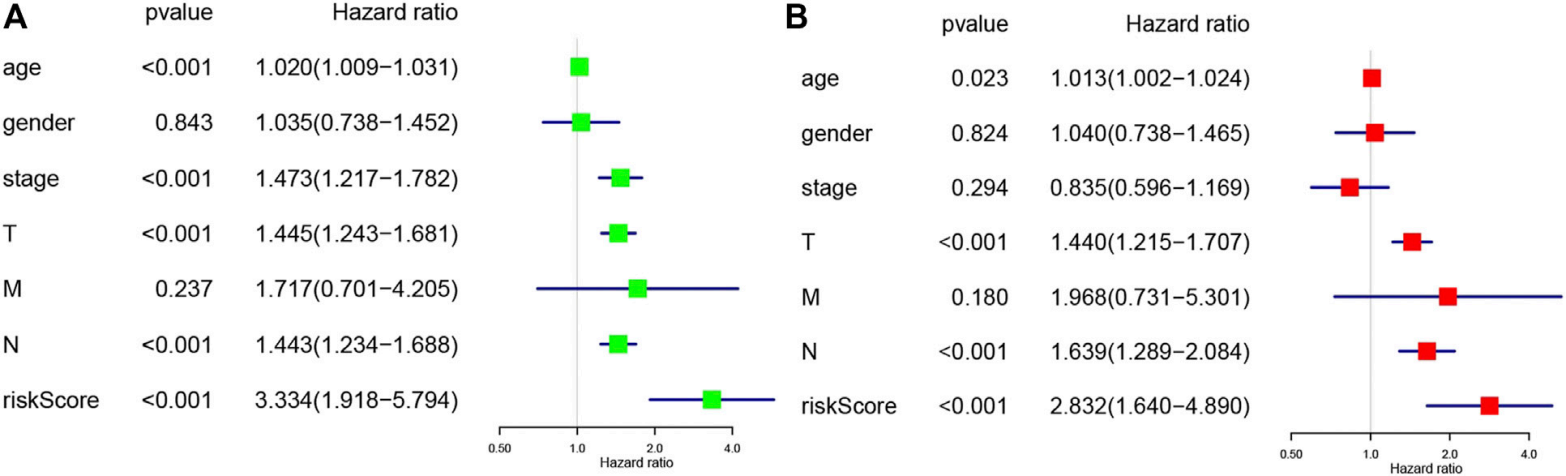
**(A)** Univariate analysis of the hazard ratios for risk score as independent prognostic elements to aniticipate the overall survival. **(B)** Multivariate analysis of the hazard ratios for risk score as independent prognostic elements to predict the overall survival.

Moreover, univariate (Figure 6A) and multivariate (Figure 6B) Cox analyses for overall survival were performed to estimate whether clinicopathological characteristics (including stage, sex, age, survival time, TNM and risk score) were independent prognostic factors. It has been proven that T stage, N stage, risk score, and age were all independent factors for poor prognosis in cutaneous melanoma patients. Multivariate analysis sharing the same variables with the univariate analysis which supported that T (*p* = 2.61E-05, 95% CI HR 1.215–1.707), N (*p* = 5.61E-05, 95% CI HR 1.289–2.084), risk score (*p* = 0.00019, 95% CI HR 1.640–4.890), age (*p* = 0.02298, 95% CI HR 1.002–1.024). They were also served as poor prognostic factors for cutaneous melanoma patients.

### Genomic Alterations and Clinical Implications in Cutaneous Melanoma

**FIGURE 7:**
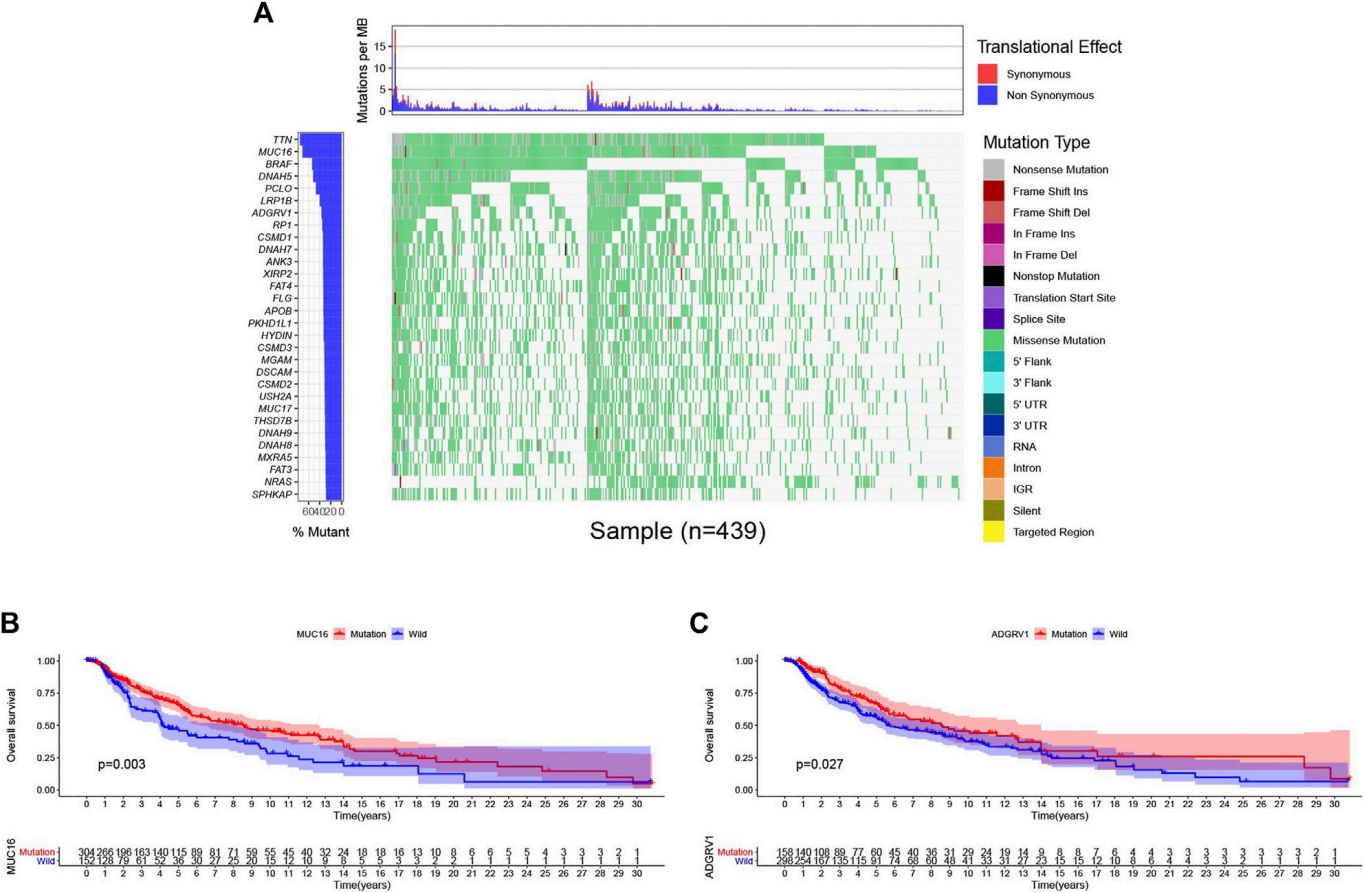
Genomic alterations and clincal implications in cutaneous melanoma. **(A)** The waterfall plots of top 30 mutated genes in TCGA database. **(B–C)** Kaplan-Meier survival analysis for MUC16 **(B)**, ADGRV1 **(C)** mutated genes.

Then, we downloaded the SNP data from the 471 samples of cutaneous melanoma and executed mutation visualization analysis through the R package “GenomeInfoDbData” and “GenVisR”. From the waterfall chart (Figure 7A) we obtained; we identified the top 30 mutated genes in the 439 samples. The mutation rates of TTN and MUC16 were over 60%. Kaplan-Meier analysis was used to detect these top 30 mutant genes. Only MUC16(Figure 7B) and ADGRV1 (Figure 7C) showed significant differences (*p* < 0.05) in overall survival between the mutant and wild type.

**FIGURE 8:**
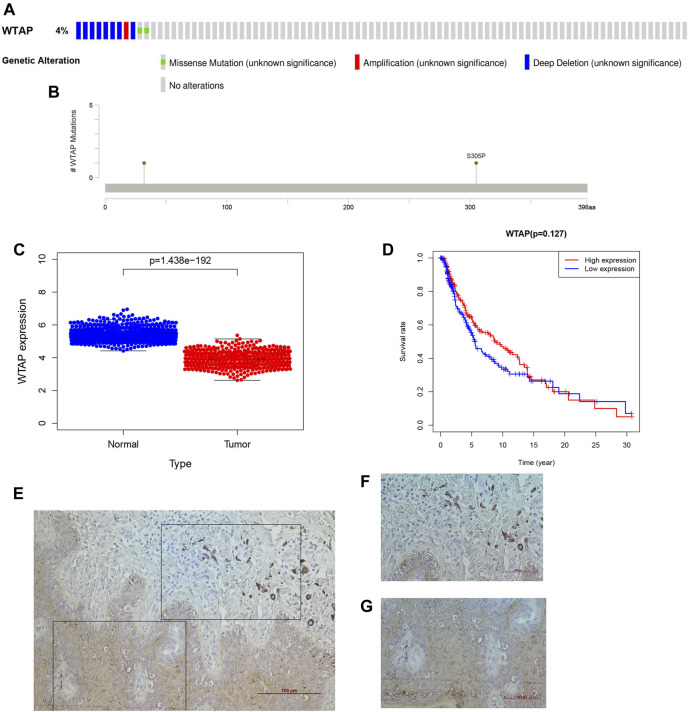
WTAP as a novel prognostic biomarker in cutaneous melanoma. **(A)** OncoPrint of WTAP altertions in melanoma cohort identified by cBioPortal. **(B)** lollipop of WTAP alteration in melanoma cohort identified. **(C)** The differential expression of WTAP in cutaneous melanoma. **(D)** The overall survival of cutaneous melanoma patients in the two clusters (high WTAP expression group and the low WTAP expreaaion group) was calculated by Kaplan-Meier curves. **(E-G)** The histological features of WTAP gene in cutaneous melanoma **(F)** and their adjacent tissuses **(G)**.

Given that the m^6^A RNA methylation regulator-based signature was closely related to tumor stage and T stage in TNM staging, we wanted to further evaluate those genes that can be treated as independent prognostic factors. Wilms’ tumor 1-associating protein (WTAP) functions as one of the main components of the mA methyltransferase complex in humans. Moreover, a complementary study (Ping et al., 2014) demonstrated that the RNA-binding capability of METTL3 is obviously inhibited when WTAP is absent. WTAP may regulate the activity of its m^6^A methyltransferase complex toward mRNA targets. Thus, we subsequently focused on the m^6^A writer WTAP. First, we used cBioPortal to identify the frequency and location of WTAP mutations. According to Oncoprint (Figure 8A), WTAP is altered in 11 (4%) of the examined patients, including cases of missense mutation, amplification, and deep deletion. Among them, deep deletion accounts for the vast majority of alterations. A lollipop diagram of WTAP was generated to show the locations of the gene mutations (Figure 8B). Next, we arranged the transcribed data into two groups, depending on the mutation data in cBioPortal. A total of 469 samples belonged to the wild-type group, while only two samples belonged to the mutation group. We screened the differential expression of the genes between the two groups through logFC > 1 and FDR < 0.05. Moreover, the heatmap (Supplementary figure 2A) and the volcano map (Supplementary figure 2B) may show the difference more obviously. Only red dots appear in the volcano map, which means that the WTAP alteration enhances the expression of those genes. To better understand the functions of the differentially expressed genes between the two groups, we performed Gene Ontology (GO) enrichment analysis. The results showed that the differentially expressed genes mainly function in the “immunoglobin complex,” “phagocytosis,” and “antigen binding” categories in the cellular component (CC) (Supplementary figure 2C), molecular function (MF) (Supplementary figure 2D), and biological process (BP) categories (Supplementary figure 2E), respectively. KEGG enrichment analysis (Supplementary figure 2F) revealed significant enrichment of genes in the “GABAergic synapse,” “morphine addiction,” “serotonergic synapse” and “retrograde endocannabinoid signaling” pathways. The protein-protein interaction (PPI) (Supplementary figure 2G) network analysis indicated that ALDH1A3 and ACSS2 had the most interactions of the studied genes.

### WTAP in Cutaneous Melanoma and the Prediction of the Downstream Signaling Pathway

WTAP is recognized as an important writer of the m^6^A RNA methylation transferase complex. As deduced from the above, it is also a protective factor for the development of cutaneous melanoma. To better understand the influence of WTAP on clinically related factors in cutaneous melanoma and whether it can be treated as an independent prognostic factor to judge the prognosis of cutaneous melanoma, we performed a series of bioinformatic analyses. In the figure, we can see that there was an obvious difference in expression between the normal tissue and the tumor group (Figure 8C), and the observed attenuation of WTAP expression in the tumor group also proved its protective role. Then, two groups were formed through the expression level of WTAP in the sample, and Kaplan–Meier analysis with the log-rank test was also applied to judge the difference in overall survival (OS) between the high- and low-expression groups (Figure 8D). Here, there was a difference, but it was not statistically significant. We went a further step to verify the roles of WTAP in melanoma, we first examined the expression of WTAP in melanoma and adjacent tissues. It was noteworthy that the expression of the known m^6^A “writer” WTAP was significantly declined in cutaneous melanoma than the adjacent tissue (Figures 8E–G), which is consist with the analysis we performed.

**FIGURE 9:**
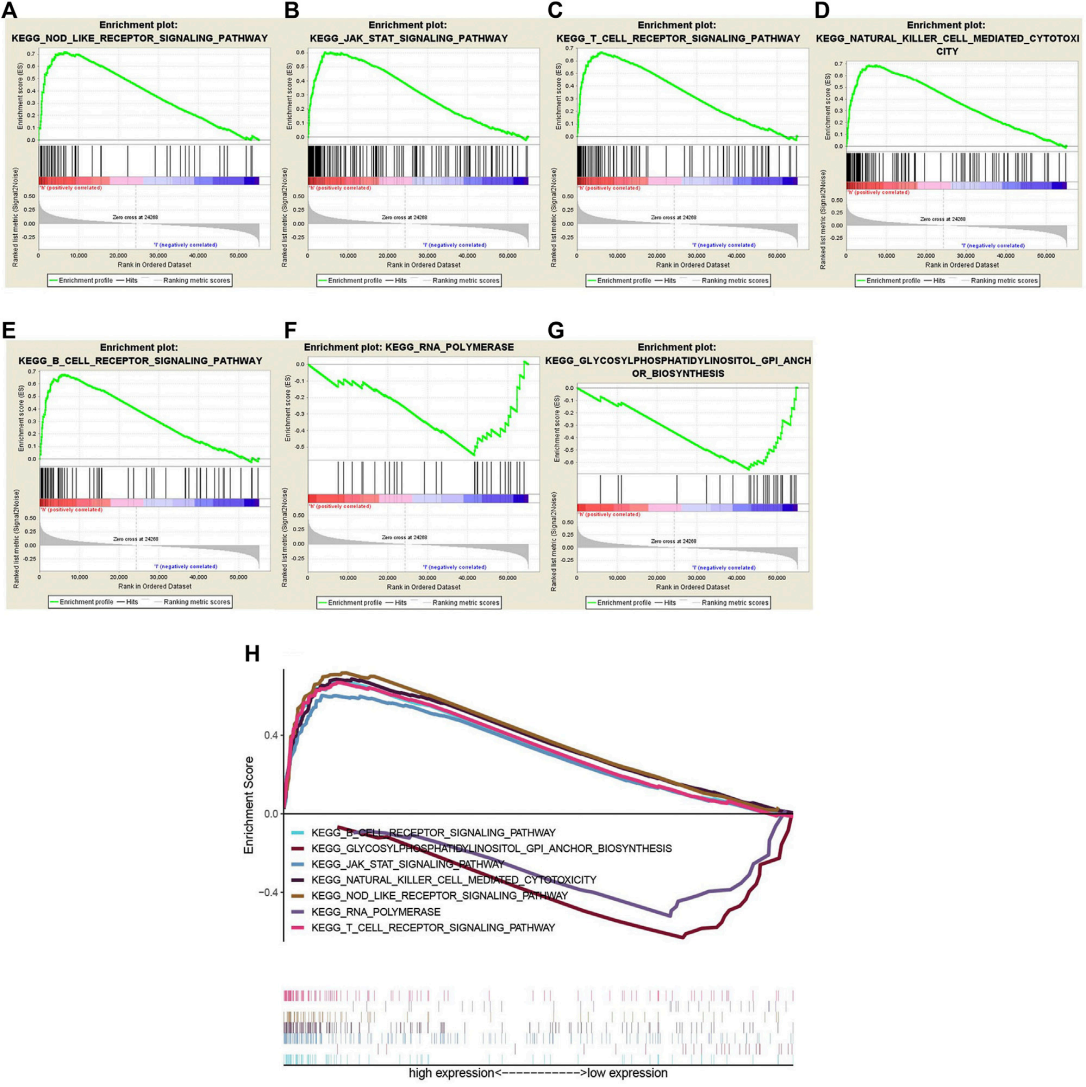
**(A–G)** Gene set enrichment analysis (GSEA) was conducted to predict the potential functions and pathways regulated by WTAP base on Kyoto Encyclopedia of Genes and Genomes (KEGG) datasets: NOD-like receptor signaling pathway **(A)**, JAK-STAT signaling pathway **(B)**, T cell receptor signaling pathway **(C)**, Natural killer cell mediated cytotoxicity **(D)**, B cell receptor signaling pathway **(E)**, RNA polymerase **(F)**, glycosylphosphatidylinositol (GPI)-anchor Biosynthesis **(G)**. **(H)** Multiple Gene Set enrichment analysis (GSEA).

GSEA was executed to predict the downstream pathway induced by WTAP, to help us to more comprehensively understand how WTAP works. FDR < 0.05 was used as the screening condition, and we obtained seven possible pathways. Among them, the pathways that were positively correlated with WTAP gene expression included “NOD-like receptor signaling pathway (Figure 9A), JAK-STAT signaling pathway (Figure 9B), T cell receptor signaling pathway (Figure 9C), Natural killer cell mediated cytotoxicity (Figure 9D), B cell receptor signaling pathway (Figure 9E), RNA polymerase (F), Glycosylphosphatidylinositol (GPI)-anchor Biosynthesis (Figure 9G)”. Through multiple GSEA (Figure 9H), we summarized the results into one graph.

### Validation the Effect of WTAP in Cutaneous Melanoma Tissues and Cells

**FIGURE 10:**
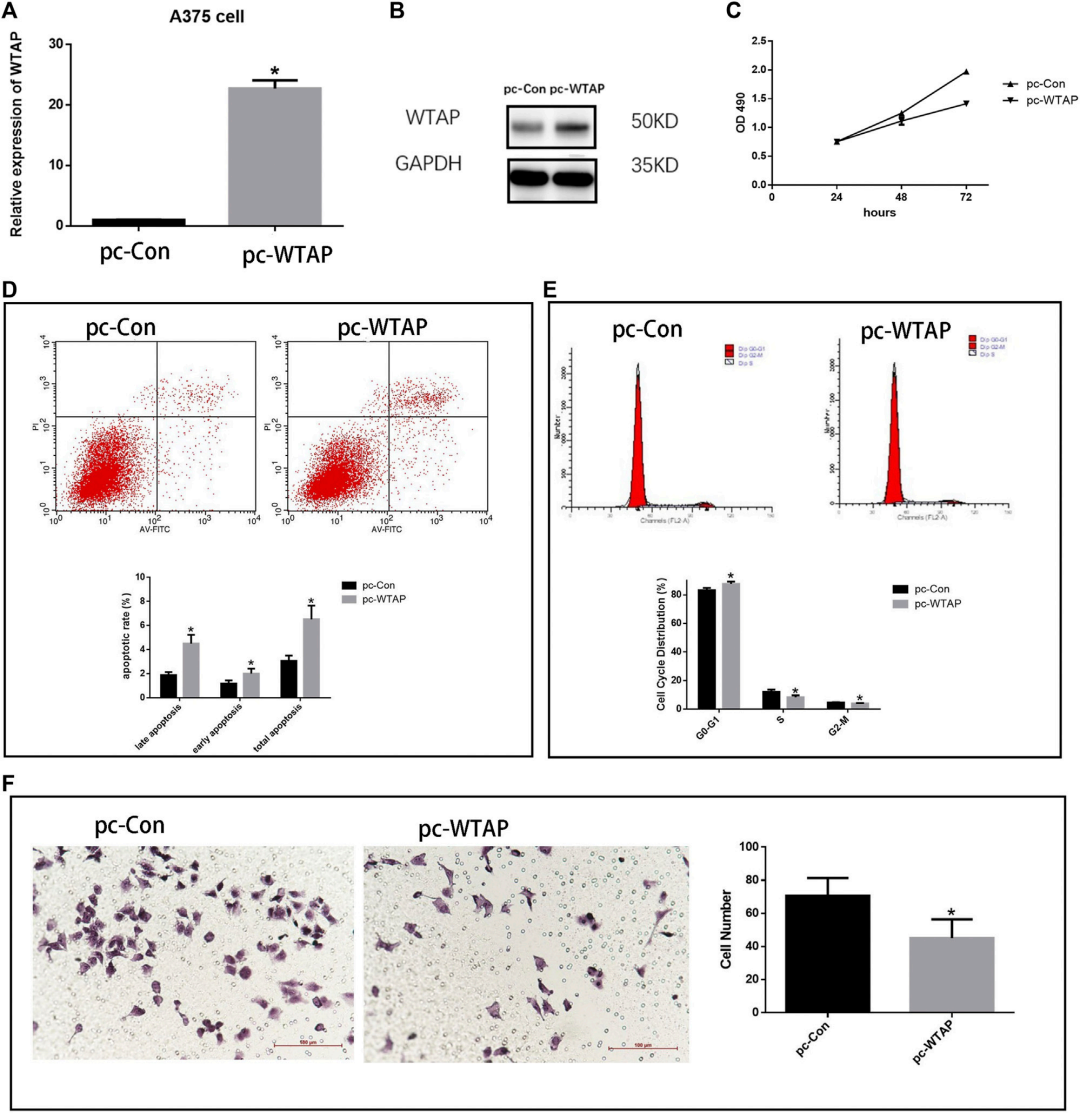
Overexpression WTAP inhibited the aggressive behaviors of melanoma cells. **(A)** Plasmids transfected into A375 cells to overexpress the WTAP expression. The mRNA level of WTAP in A375 cell lines was detected by qRT-PCR. **(B)** The expression of WTAP in A375 cell lines was detected by western blotting **(C)** The proliferation of cells was detected by cell counting Kit-8 (CCK-8). **(D)** The apoptosis of A375 cells with WTAP overexpression **(E)** cell cycle of A375 cells was evaluated by flow cytometry. **(F)** Migration of A375 cells were detected by transwell assay.

To further validate the effect of WTAP in cutaneous melanoma, plasmids were transfected into melanoma cell line A375 cells to overexpress WTAP. Quantitative real-time PCR (Figure 10A) and Western Blotting (Figure 10B) proved the success of transfection. The proliferation of melanoma cells was significantly repressed in the overexpressed group (Figure 10C). Consistently, in apoptosis assay, the apoptotic rate of A375 cells was significantly increased while both early apoptosis and late apoptosis are promoted in WTAP overexpressed group (Figure 10D). In cell cycle assay, overexpression of WTAP upregulated the proportion of cells in G0-G1 stage, while downregulated the proportion of cells in S stage (Figure 10E). Furthermore, the migration assays indicated the migration was inhibited in the WTAP overexpressed group (Figure 10F). These results indicated that WTAP is a protective gene in melanoma.

## Discussion

In recent years, abundant genome-wide studies have proven that the majority of the genes are transcribed, thus forming an RNA network consisting of large and small RNAs in cells. However, only a small number of transcripts are translated to proteins (Pandiani et al., 2021). Posttranscriptional RNA modification is an essential process for translation. An estimated approximately 150 posttranscriptional RNA modifications have been proven in all species (Ogawa et al., 2021). Among those modifications, m^6^A is the most popular among eukaryotic mRNAs and long noncoding RNAs (Kwok et al., 2017). It has also been indicated that anomalous expression of m^6^A methylation regulators is closely related to the diverse processes in human cancer (Yang et al., 2019; Bian et al., 2020; Ji et al., 2020). Although there have been many studies on the function of m^6^A RNA methylation regulators in various types of cancer and the related pathways through which they exert their function, research in the field of cutaneous melanoma is quite limited, and only a few studies have studied uveal melanoma (Tang et al., 2020) and ocular melanoma (Jia et al., 2019).

Cutaneous melanoma is still the leading cause of cutaneous cancer-related deaths (Gupta et al., 2020). The treatment of cutaneous melanoma is complex and involves multidisciplinary methods, including surgery, adjuvant radiation and new systemic treatments, such as targeted agents and immunotherapy (Nenclares et al., 2020). Although its incidence rate is low, it is highly malignant (Ablain et al., 2018), metastasis occurs early, and its mortality is high, so it is necessary to study cutaneous melanoma systematically.

In our research, we examined the expression levels of 17 frequently mentioned m^6^A RNA methylation regulators in the TCGA-SKCM dataset. It was demonstrated that there is a large difference between normal and tumor tissue. We then separated the melanoma cohort into two clusters through consensus clustering analysis. The survival curve after clustering did not show significant statistical significance, which may be due to the insufficient number of samples or clustering needs to be adjusted.

According to the results of the multivariate Cox regression analysis, three of the seventeen m^6^A RNA methylation regulators were proved to be the potential prognostic factors of cutaneous melanoma, including ELF3, ZC3H13, and WTAP.

The LASSO algorithm was applied with the three mentioned genes to build a risk signature. The signature risk score was associated with different sizes of primary tumors, which could also be treated as an independent prognostic factor. Among the 17 m^6^A regulators, previous studies have reported that high WTAP expression in hepatocellular carcinoma was associated with poor prognosis; hence, WTAP promoted the growth and proliferation of HCC cells both *in vitro* and *in vivo* (Chen et al., 2019). Moreover, in bladder cancer patients, the expression level of WTAP is closely related to the risk of recurrence (Chen and Wang, 2018). In high-grade serous ovarian carcinoma, high expression of WTAP correlated with shorter overall survival, and low expression of WTAP reduced cancer cell proliferation and migration (Yu et al., 2019). Meanwhile, the reader ELF3 was reported to be closely related to poor survival in colorectal cancer (CRC) patients by driving β-catenin transactivation (Wang et al., 2014). Thus, m^6^A regulators are closely correlated with the prognosis of cancer. In our paper, we report further research on the methylation writer WTAP.

To analyze the mutation profile of cutaneous melanoma in the TCGA dataset, we identified the top 30 variant mutated genes. Difference analysis and functional enrichment analysis, as well as the construction of a PPI network, helped us to have a better understanding of its functions and GO annotations of genes involved in cutaneous melanoma. Then, we focused on WTAP in TCGA, and we performed a series of studies to detect the mutation probability and the survival rate of the mutation group and the normal group. The difference between the groups was not significant, possibly because the mutation rate was so low that we did not have enough samples in our analysis to detect a significant difference.

WTAP as a single factor was further analyzed in the tumor group and normal group, the relationship between high and low WTAP expression and clinicopathological factors, and whether it can be treated as an independent prognostic factor. Next, we tried to determine its potential mechanisms via bioinformatic prediction and molecular validation. The downstream pathways were further predicted by GSEA, with the corrected p value as the screening condition, and seven pathways were identified and selected for display. Further exploration with further experiments and analysis is needed.

## Data Availability

The datasets presented in this study can be found in online repositories. The names of the repository/repositories and accession number(s) can be found in the article/Supplementary Material.
