# Supplementary figures and images for: Identification of the m6A RNA Methylation Regulators WTAP as a Novel Prognostic Biomarker and Genomic Alterations in Cutaneous Melanoma
Source: Front Mol Biosci. 2021 Jul 5;8:665222. doi: 10.3389/fmolb.2021.665222 (PMC8287526; doi:10.3389/fmolb.2021.665222)

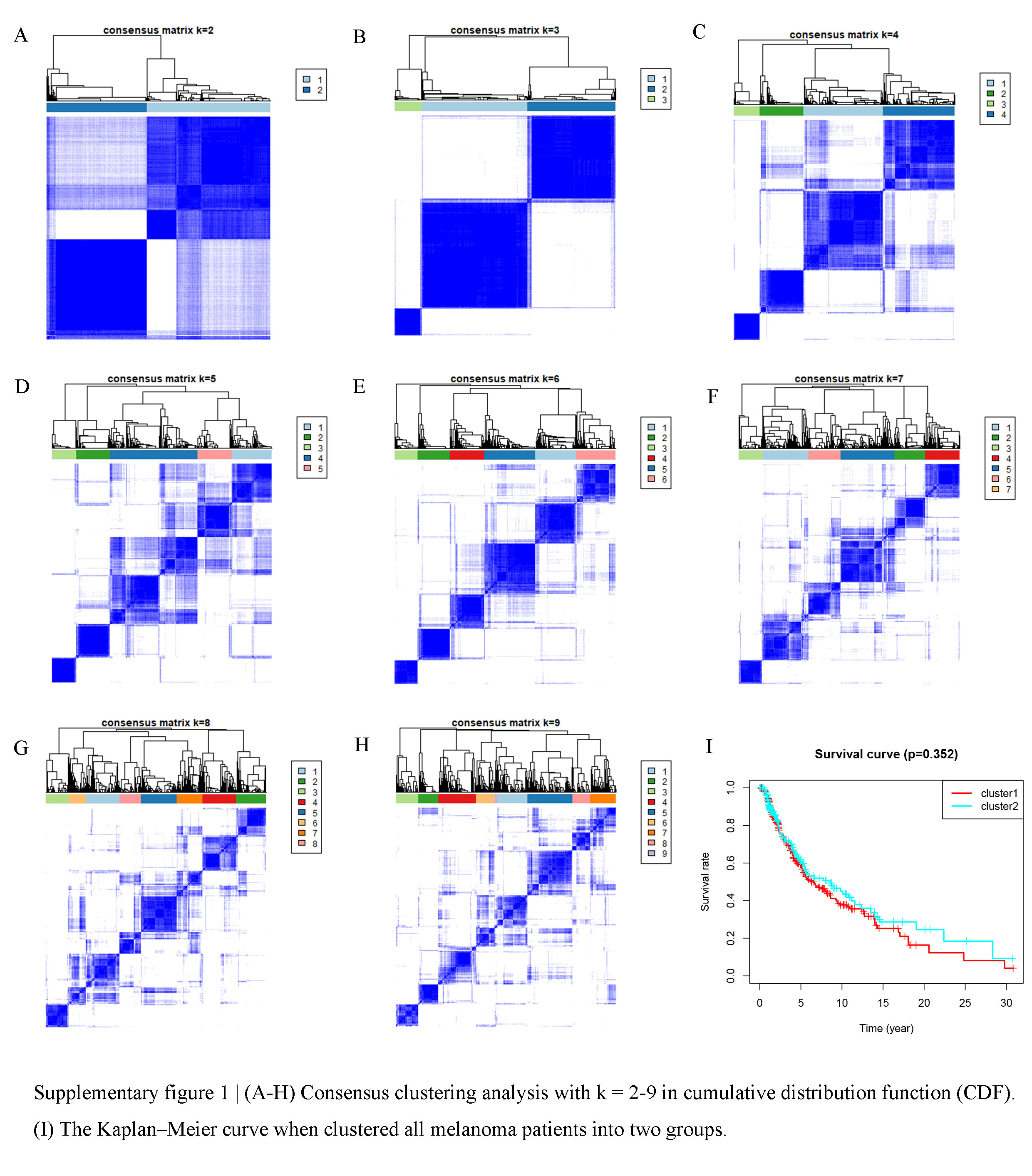

Supplement: Supplementary file 1 [file Image1.JPEG]

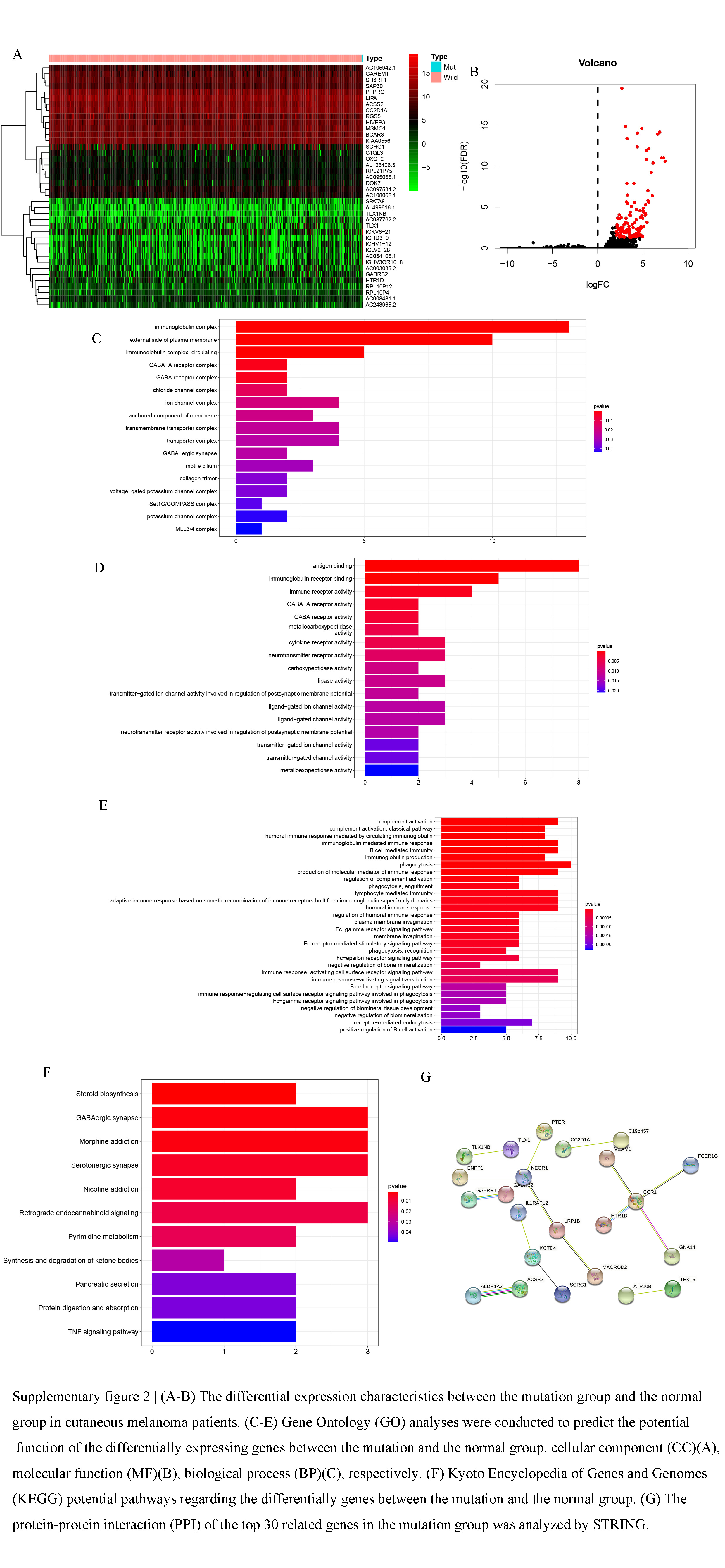

Supplement: Supplementary file 2 [file Image2.JPEG]
